# NRF1 and NRF2 mRNA and Protein Expression Decrease Early during Melanoma Carcinogenesis: An Insight into Survival and MicroRNAs

**DOI:** 10.1155/2019/2647068

**Published:** 2019-09-04

**Authors:** Mari Hämäläinen, Hanna-Riikka Teppo, Sini Skarp, Kirsi-Maria Haapasaari, Katja Porvari, Katri Vuopala, Thomas Kietzmann, Peeter Karihtala

**Affiliations:** ^1^Cancer Research and Translational Medicine Research Unit, University of Oulu, Oulu University Hospital and University of Oulu, Oulu, Finland; ^2^Department of Oncology and Radiotherapy, Medical Research Center Oulu, Oulu University Hospital and University of Oulu, Oulu, Finland; ^3^Infrastructure for Population Studies, Faculty of Medicine, University of Oulu, Oulu, Finland; ^4^Department of Pathology, Lapland Central Hospital, Rovaniemi, Finland; ^5^Faculty of Biochemistry and Molecular Medicine, Biocenter Oulu, University of Oulu, Oulu, Finland

## Abstract

The prognostic significance of the major redox regulator nuclear factor erythroid-2-related factor (NRF2) is recognized in many cancers, but the role of NRF1 is not generally well understood in cancer. Our aim was to investigate these redox transcription factors in conjunction with redox-related microRNAs in naevi and melanoma. We characterized the immunohistochemical expression of NRF1 and NRF2 in 99 naevi, 88 primary skin melanomas, and 67 lymph node metastases. In addition, NRF1 and NRF2 mRNA and miR-23B, miR-93, miR-144, miR-212, miR-340, miR-383, and miR-510 levels were analysed with real-time qPCR from 54 paraffin-embedded naevi and melanoma samples. The immunohistochemical expression of nuclear NRF1 decreased from benign to dysplastic naevi (*p* < 0.001) and to primary melanoma (*p* < 0.001) and from primary melanoma to metastatic lesions (*p* = 0.012). Also, NRF1 mRNA levels decreased from benign naevi to dysplastic naevi (*p* = 0.034). Similarly, immunopositivity of NRF2 decreased from benign to dysplastic naevi (*p* = 0.02) and to primary lesions (*p* = 0.018). NRF2 mRNA decreased from benign to dysplastic naevi and primary melanomas (*p* = 0.012). Analysis from the Gene Expression Omnibus datasets supported the mRNA findings. High nuclear immunohistochemical NRF1 expression in pigment cells associated with a worse survival (*p* = 0.048) in patients with N0 disease at the time of diagnosis, and high nuclear NRF2 expression in pigment cells associated with a worse survival (*p* = 0.033) in patients with M0 disease at the time of diagnosis. In multivariate analysis, neither of these variables exceeded the prognostic power of Breslow. The levels of miR-144 and miR-212 associated positively with ulceration (*p* = 0.012 and *p* = 0.027, respectively) while miR-510 levels associated positively with lymph node metastases at the time of diagnosis (*p* = 0.004). Furthermore, the miRNAs correlated negatively with the immunohistochemical expression of NRF1 and NRF2 but positively with their respective mRNA. Together, this data sheds new light about NFE2L family factors in pigment tumors and suggests that these factors are worth for further explorations.

## 1. Introduction

Nuclear factor erythroid-2-related factor 2 (NRF2) is the most studied member of the Cap ‘n' collar basic leucine zipper (CNC-bZIP) family of transcription factors. It is a main inductor of genes of antioxidant proteins and phase II detoxifying enzymes [1]. In addition, due to activating mutations, growth signalling and epigenetic dysregulation NRF2 was also found be aberrantly activated in several cancers [2, 3]. From the same family of transcription factors, NRF1 is generally far less studied and its role in carcinogenesis is insufficiently explored. Similar to NRF2, it is responsive to oxidative stress and activates antioxidant responsive element- (ARE-) driven genes [4]. Both, NRF1 and NRF2 reside outside of the nucleus under unstressed conditions: NRF1 in the endoplasmic reticulum (ER) and NRF2 in the cytoplasm [5]. Several events contribute to NRF1 and NRF2 activation, among them the proteolytic cleavage of NRF1 from the ER membrane and the phosphorylation of NRF2. As a consequence, both factors are transported to the nucleus to induce the expression of their target genes.

MicroRNAs (miRNAs) are small noncoding RNAs that posttranscriptionally regulate gene expression by imperfect matching of mRNA [6]. The so-called redoximiRs represent an additional regulatory mechanism for redox homeostasis. In particular, miR-23B, miR-93, miR-144, and miR-212 were found to play a role as NRF2 inhibitors, while miR-340 appears to have a role as an NRF1 and MAPK inhibitor with miR-383 and miR-510 having a less clear role in the regulation of NRF1 and NRF2 levels [7]. Furthermore, it has been shown that miRNAs have a substantial role in melanocyte and melanoma biology [8] and that they affect, for instance, melanoma cell proliferation, invasion, and migration [9]. A total of 63 differentially expressed miRNAs have been previously linked to metastatic melanoma, many of which are known to be associated with multiple different cancers [10]. Previous studies also show that miRNA expression differs in healthy patients as compared to patients with melanoma and that miRNA expression associates with patient survival rate. All in all, miRNAs could be used as potential diagnostic, prognostic, and predictive markers in the future [11].

We have previously described the expression and prognostic role of the NRF2 immunohistochemical expression in primary and metastatic melanoma [12, 13]. Here, we have extended those studies and investigated the activated state of both factors in an enlarged sample set of naevi and melanoma. To do this, we explored active NRF2 with a phosphorylation-specific antibody [14] and NRF1 with two different antibodies targeting its N- and C-terminal domains to reflect its inactive and active location and activation, respectively [5]. In addition, NRF1 and NRF2 mRNAs and the redox-related miRNAs miR-23B, miR-93, miR-144, miR-212, miR-340, miR-383, and miR-510 were analysed from the same material and three Gene Expression Omnibus (GEO) datasets, and the results were correlated to the clinical and histopathological factors.

## 2. Materials and Methods

The study included 172 patients and 255 patient samples (Table 1) collected from the paraffin block archives stored in the Department of Pathology at Oulu University Hospital between 2001 and 2016 and in the Department of Pathology at Lapland Central Hospital between 2010 and 2016. All samples were fixed in neutral buffered formalin and embedded in paraffin. Cases were randomly collected based on the diagnosis and the adequacy of the samples for RNA extraction. The series consisted of 53 benign naevi (25 compositus, 28 intradermal), 46 dysplastic naevi, 48 nodular melanomas, 32 superficially spreading melanomas, and 9 acral melanomas. Out of all malignant samples, 59 were metastatic melanomas with, respectively, 67 lymph node metastases available (one or several per case). All samples were used for immunohistochemical analysis, but for RNA isolation and qPCR analysis, only selected cases were included based on the estimated sufficiency of the tumorous tissue (*n* = 54, Table 1). Diagnoses were according to the current WHO classification. Clinical data and pathologists' reports of the cases were collected retrospectively from the patient records of Oulu University Hospital and Lapland Central Hospital. We also collected data on adjuvant therapy or treatment at a possible metastatic stage, but only a few patients received oncological treatments, and therefore, no statistical analyses on the predictive power of the markers were able to be used.

### 2.1. NRF1 and NRF2 Immunohistochemistry

Sections of 3-4 *μ*m thickness were cut from samples routinely fixed in formalin and embedded in paraffin. Tissue sections were deparaffinised in xylene (2 min, 4 times) and rehydrated through graded ethanol. Antigen retrieval was performed according to Table 2 by boiling with microwaves at 95°C for either 12 minutes (sodium citrate buffer) or 20 minutes (TrisEDTA). After boiling, the sections were allowed to cool at room temperature (RT) and washed using PBS 3 times. The sections were incubated in 3% hydrogen peroxide for 5 minutes to inactivate endogenous peroxidases. After washing repeatedly by PBS for 5 minutes, sections were incubated with the primary antibody (Table 2), then washed repeatedly by PBS for 5 minutes and incubated with a secondary antigen retrieval system at RT (Table 2). After washing repeatedly by PBS, the labelled secondary antibody was visualised according to the manufacturer's instructions. Sections were then counterstained with haematoxylin, dehydrated, and mounted. To evaluate the immunohistochemical data, the staining intensity was evaluated from the tumorous cells as one of the following expressions: negative, weak positive, or strong positive. The quantity of each intensity level was recorded (0-100%). Subsequently, a modified Histoscore was used with the following algorithm: 0 × negative expression percentage + 1 × weak expression percentage + 3 × strong expression percentage (range 0-300).

### 2.2. RNA Isolation and qPCR Analysis

Tumorous tissue was macrodissected from 2 to 6 sections of 10 *μ*m thickness. Samples were estimated to represent melanocytic proliferation for >80% of the volume. Macrodissected samples were collected into Eppendorf tubes and deparaffinised using deparaffinisation solution (Qiagen, Hilden, Germany), and total RNA was extracted from paraffin samples using the miRNeasy FFPE Kit (Qiagen). cDNA synthesis was done using the miScript II Reverse Transcription Kit (Qiagen). The miScript SYBR Green PCR Kit (Qiagen) was used for cDNA amplification by the Rotor-Gene Q real-time quantitative PCR equipment (Qiagen). Amplicon length was checked by gel electrophoresis.

**Figure 1:**
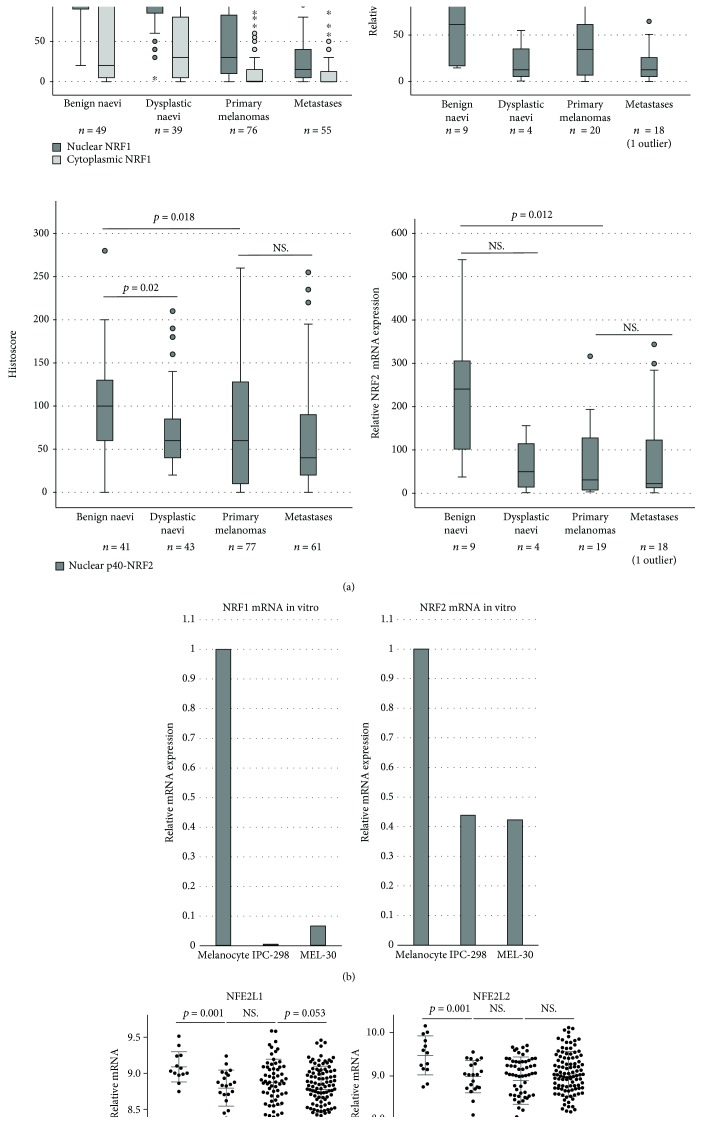
Immunohistochemical and mRNA expression of NRF1 and NRF2. (a) Boxplots representing the Histoscore of the immunohistochemical expression of NRF1 and p40-NRF2 and relative mRNA levels of NRF1 and NRF2 from paraffin-embedded patient samples, (b) expression levels of NRF1 and NRF2 mRNA from representative cell culture samples, and (c) Pooled GEO data from three different cDNA microarray studies including the expression levels of NFE2L1 and NFE2L2 (NRF1 and NRF2). Outliers of the figures are reported.

For mRNA and miRNA quantification, both specially designed (Sigma) and commercial miScript Primer Assays (Qiagen) were used for amplification, respectively (Table 3). GAPDH mRNA and miScript Primer Assay for RNU6B were used for normalization of qPCR results. Cycling was carried out as recommended in the PCR Kit with annealing temperatures of 60–68°C or 55°C for mRNA and miRNA, respectively. Fluorescence signals were measured continuously during repetitive cycles to detect Ct values for target RNA and reference (GAPDH or RNU6B) in the samples. Relative expression levels of mRNA or miRNA targets were calculated using the 2^−*ΔΔ*Ct^ method [15], where ΔΔCt = (Ct_target RNA_‐Ct_GAPDH or RNU6B_)_sample_‐(Ct_target RNA_‐Ct_GAPDH or RNU6B_)_reference sample_. Representative cell culture samples were run and analysed in parallel with the patient samples (Figure 1(b)). RNA from melanocytes was used as a reference sample (with a given value of 1) in relative expression level calculations.

### 2.3. GEO Datasets

The three microarray datasets GSE8401, GSE46517, and GSE53223 first described in original articles [16–18] were downloaded in .CEL-file format from the Gene Expression Omnibus (National Center for Biotechnology Information). Data was analysed using Chipster v3.14 software [19]. Datasets were first normalized individually and then, when combining the datasets, the batch effect was corrected using ComBat. The differential mRNA expression levels of NFE2L1 and NFE2L2 (NRF1 and NRF2) were determined and tested with the empirical Bayes *T*-test between the diagnostical groups. The combined data contained normal skin samples (*n* = 13), benign and dysplastic naevi (*n* = 21), primary melanoma lesions (*n* = 62), and metastatic melanoma lesions (*n* = 104). The respective results were plotted with GraphPad Prism 7.05.

### 2.4. Cell Lines

Cell lines representing human primary melanoma IPC-298 (ACC 251), metastatic melanoma SK-MEL-30 (ACC 151), and adult primary epidermal melanocytes (PCS-200-013) were ordered from Leibniz-Institut, DSMZ (Braunschweig, Germany) and ATCC (LGC Standards GmbH, Germany). Melanoma cells were cultured in RPMI-1640 with 10% foetal bovine serum and 100 IU/ml penicillin and streptomycin (Pen-Strep solution HyClone laboratories Inc., UT, USA). Melanocytes were cultured in a Dermal Cell Basal Medium supplemented with an Adult Melanocyte Growth Kit (PCS-200-030 and PCS-200-042 from ATCC). Cells were cultured in 37°C 5% CO_2_.

### 2.5. Western Blot Analysis

The fractionated lysates were prepared by using the Subcellular Protein Fractionation Kit for Cultured Cells (Thermo Scientific, IL, USA). Protein concentrations were measured using the Bio-Rad Protein Assay (Bio-Rad; CA, USA), and the concentration in individual samples was equalized before adding 4x Laemmli buffer to a final concentration of 1x. Equal amounts of protein were run on 7.5% SDS-PAGE gels, transferred to PVDF membranes, probed with the antibodies (Table 2), and diluted with 5% bovine serum albumin in tris-buffered saline with 0.1% Tween 20. Primary antibodies were incubated overnight, and appropriate HRP-conjugated secondary antibodies were incubated at RT for one hour (Table 2). Blots were detected with the ECL chemiluminescence system (Pierce ECL Western Blotting Substrate, Thermo Scientific, IL, USA) on radiographic films, which were then scanned to an electronic format.

### 2.6. Statistical Analyses

Statistical analyses were performed by using IBM SPSS Statistics software, v. 25.0.0.0 (IBM Corporation, Armonk, NY, USA). The significance of associations was defined by using the Mann–Whitney *U* test and Spearman's rho test with a correlation coefficient. The Kaplan-Meier curves with the log-rank test were applied in survival analyses, along with Cox regression to perform multivariate analysis. In determining a two-classed variable for survival analysis, a Histoscore cut-off value (32.5) was chosen using a Receiver Operating Characteristic Curve (ROC) analysis for NRF1, the highest Histoscore quartal for NRF2, and the median for mRNA and miRNA levels. Disease-specific survival (DSS) was calculated from the time of diagnosis to the time of confirmed melanoma-related death. Values of *p* of less than 0.05 were considered significant.

### 2.7. Ethical Approval

The study was approved by Valvira, the Finnish National Supervisory Authority for Welfare and Health, and the Local Ethics Committee of the Northern Ostrobothnia Hospital District. During data collection and management, the principles of the Helsinki Declaration were followed. The authors declare that they have no competing interests and that funding sources had no involvement in the study.

## 3. Results

### 3.1. Immunohistochemical and mRNA Expression of NRF1 and NRF2 in Naevi, Primary Melanomas, and Melanoma Metastases and Their Association with Histopathological and Clinical Parameters

**Figure 2:**
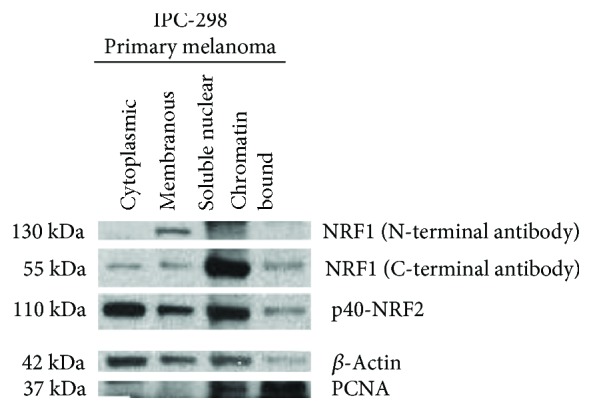
Protein expression of NRF1 and p40-NRF2 in western blot, fractionated cell lysates from IPC-298 melanoma cells, *β*-actin, and PCNA (proliferating cell nuclear antigen) serve as loading and fractioning controls. NRF1 detected with an antibody targeting N-terminal domain is expressed in membranous fraction, and NRF1 detected with an antibody targeting C-terminal domain is expressed mostly in the nuclear fraction. p40-NRF2 is expressed in the cytoplasmic, membranous, and nuclear fractions.

First, we tested whether the antibodies against the N-terminal domain of NRF1 and the C-terminus of NRF1 as well as against phosphorylated NRF2 detect the respective localization and activity status of the proteins. To do this, we performed western blot analyses where we detected the proteins in respective subcellular fractions of primary melanomas. According to expectations, the antibody detecting the N-terminal domain of NRF1, i.e., the inactive ER localized protein, displayed NRF1 in the membranous fraction, whereas the antibody against the C-terminus primarily detecting the active protein showed positive staining in the nuclear fraction. Active NRF2 (p40-NRF2) was detected in all fractions, in line with the fact that its activation by phosphorylation can occur outside the nucleus (Figure 2). Thus, these data indicate that the antibodies are suitable to detect NRF1 and NRF2 by immunohistochemistry in patient samples.

**Figure 3:**
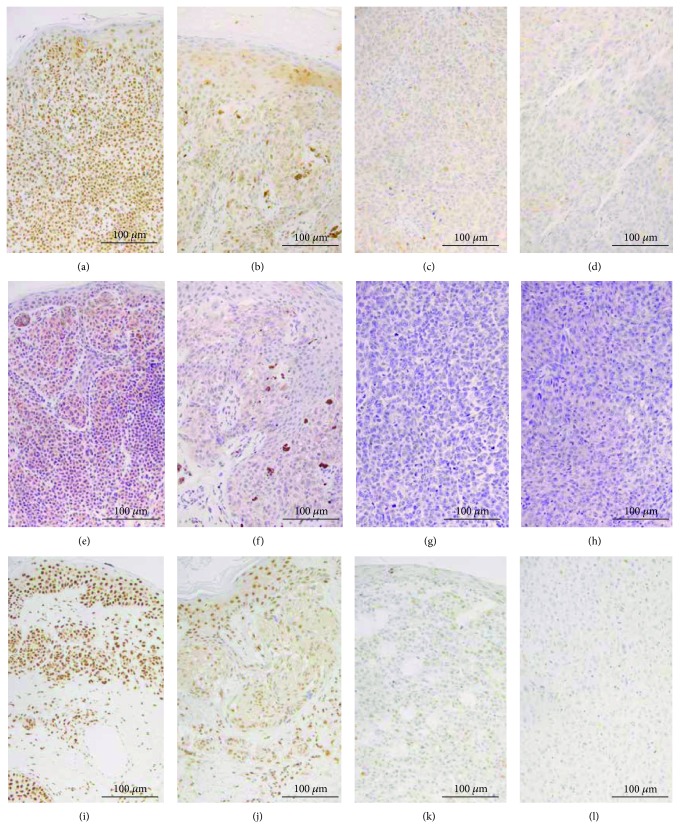
Immunohistochemical expression in patient samples. (a-d) NRF1 C-terminal antibody, benign naevus, dysplastic naevus, primary melanoma, and metastatic melanoma from a lymph node, respectively, diaminobenzidine and haematoxylin, (e-h) NRF1 N-terminal antibody, benign naevus, dysplastic naevus, primary melanoma, and metastatic melanoma from a lymph node, respectively, Fast Red and haematoxylin, and (i-l) p40-NRF2, benign naevus, dysplastic naevus, primary melanoma, and metastatic melanoma from a lymph node, respectively, diaminobenzidine and haematoxylin.

The immunohistochemistry revealed that, in line with western blots and according to the activity status, the antibody against the N-terminal domain of NRF1 detected the protein in the cytoplasm around the nucleus and never in the nuclei. The antibody against the C-terminal domain detected the protein mostly in the nuclei and rarely in the cytoplasm. The expression of both NRF1 antibodies showed a significant NRF1 decrease from benign to dysplastic naevi (*p* < 0.001 and *p* = 0.034, Supplementary [Supplementary-material supplementary-material-1], Figures 1(a) and 3) and from naevi to primary melanoma, as well as to metastatic lesions (*p* < 0.001, Figures 1(a) and 3) [20]. Nuclear NRF1 further decreased from primary to metastatic lesions (*p* = 0.012, Figures 1(a) and 3). Similarly, NRF1 mRNA levels were decreased from benign to dysplastic naevi (*p* < 0.001 and *p* = 0.034, Supplementary [Supplementary-material supplementary-material-1] and Figure 1(a)) but not from primary to metastatic lesions.

Immunopositivity of p40-NRF2 was detected mainly in the nuclei, and its expression decreased from benign to dysplastic naevi (*p* = 0.02) and then further to primary lesions (*p* = 0.018, Figures 1(a) and 3). The p40-NRF2 expression had a notable intersample variation in primary melanomas. The levels of NRF2 mRNA decreased from its highest levels in benign naevi to intermediate levels in dysplastic naevi, and its lowest levels occurred in primary melanomas (*p* = 0.012). The decrease of p40-NRF2 immunopositivity and NRF2 mRNA levels from primary to metastatic lesions was not considered significant. In addition, the decline in NRF1 and NRF2 mRNA levels from benign naevi to melanomas could be reproduced when comparing the NRF1 and NRF2 mRNA levels in cell culture lysates in the qPCR, showing a decrease of relative mRNA levels between benign melanocyte and malignant melanoma cells (Figure 1(b)).

The immunohistochemical expressions of NRF1 and p40-NRF2 did not associate with the melanoma patients' age, gender, lesion location, Breslow's thickness, ulceration, mitotic activity, or pigmentation. However, NRF1 and NRF2 mRNA correlated with melanoma patients' gender (higher mRNA levels in males, *p* = 0.037 and *p* = 0.017, *n* = 20 and *n* = 19, respectively), and NRF1 mRNA also correlated positively with the presence of ulceration (*p* = 0.016, *n* = 18).

### 3.2. Correlations of Immunohistochemical and mRNA Expression of NRF1 and NRF2

**Figure 4:**
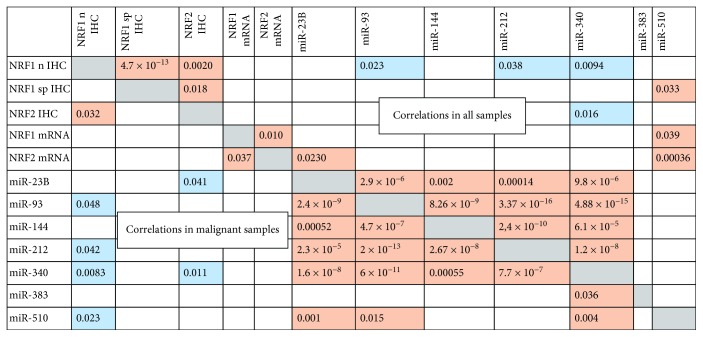
Significant correlations between studied parameters in all samples (top) and in malignant samples (bottom), *p* value. Blue box indicates negative and brown box positive correlation.

Nuclear and cytoplasmic NRF1 correlated positively in the complete series of samples (*p* = 4.7 × 10^−13^, 219 samples, Figure 4). Nuclear and cytoplasmic NRF1 and p40-NRF2 had a positive correlation in the complete series of samples (*p* = 0.0020 and *p* = 0.018, *n* = 204 and *n* = 203, respectively). Also, nuclear NRF1 and p40-NRF2 correlated positively in malignant samples (*p* = 0.032, *n* = 103, Figure 4). p40-NRF2 associated positively with our previously described NRF2 expression in the melanoma cohort with a different antibody (*p* = 0.021, *n* = 49) [12, 13]. The immunostainings did not correlate with mRNA expression levels. However, NRF1 and NRF2 mRNA expression correlated both in the complete cohort and separately in malignant samples (*p* = 0.010 and *p* = 0.037, *n* = 49 and *n* = 36, respectively, Figure 4).

### 3.3. miRNA Expression in Naevi, Primary Melanomas, and Melanoma Metastases and Association with Histopathological and Clinical Parameters

**Figure 5:**
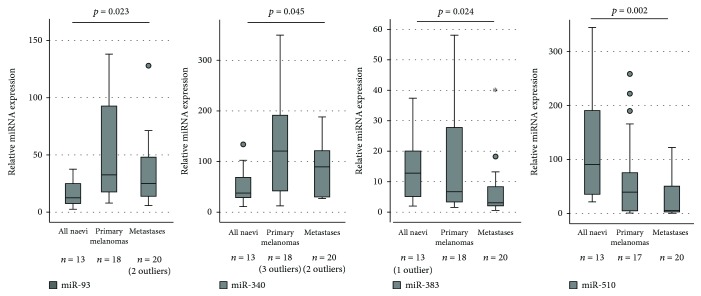
Expression levels of miR-93, miR-340, miR-383, and miR-510 in paraffin-embedded patient samples. Outliers of the figures are reported.

Significant miRNA expression alterations (Supplementary [Supplementary-material supplementary-material-1]) were not detected between benign and dysplastic naevi. However, the levels of miR-93 and miR-340 increased significantly from all naevi to primary melanomas and to metastases (*p* = 0.023 and *p* = 0.045, respectively, Figure 5). In contrast, the levels of miR-383 and miR-510 showed a decreasing trend between the three groups (*p* = 0.024, *p* = 0.002, respectively, *n* = 31, Figure 5). Moreover, significant changes in miRNA levels could not be detected between primary and metastatic melanoma lesions.

The miRNA levels did not associate with melanoma patients' gender, lesion location, or Breslow's thickness. The miR-510 levels associated positively with melanoma patients' age (*p* = 0.025, *n* = 19) and nodal disease at the time of diagnosis (*p* = 0.004, *n* = 19). In addition, the levels of miR-212 and miR-340 associated positively with pigmentation (*p* = 0.024 and *p* = 0.012, *n* = 11, respectively). Furthermore, miR-144 and miR-212 levels were found to associate positively with ulceration (*p* = 0.012 and *p* = 0.027, *n* = 18, respectively).

### 3.4. Respective Correlations of Immunohistochemical and mRNA Expression of NRF1 and NRF2 and miRNAs

There was an ample amount of significant correlation between protein and mRNA expression with different miRNAs, and this data is thoroughly presented in Supplementary [Supplementary-material supplementary-material-1] in all cases and separately in malignant samples including primary and metastatic melanoma lesions. Significant correlations between protein expression and miRNAs were always negative, except for the cytoplasmic NRF1 and miR-510, whereas correlations between mRNA and miRNAs were always positive.

### 3.5. GEO Data

Levels of NRF1 and NRF2 mRNA decreased from normal skin samples to pooled benign and dysplastic naevi (*p* = 0.001). There was no significant difference in levels between naevi and primary melanomas. Although the level of NRF1 mRNA decreased nearly significantly between primary melanomas and metastases (*p* = 0.053), the difference in NRF2 mRNA levels was not significant between primary melanomas and metastases (Figure 1(c)).

### 3.6. Survival and Cox Regression Analysis

**Figure 6:**
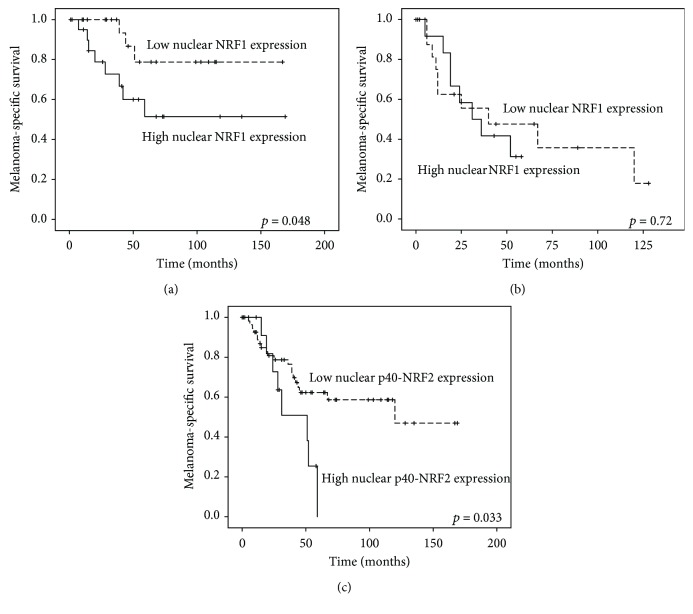
(a) High nuclear NRF1 expression (cut-off 32.5 based on ROC analysis) associated with worse melanoma-specific survival in those patients without nodal metastases at the time of diagnosis. (b) NRF1 did not separate the groups in cases with nodal metastases at the time of diagnosis. (c) High nuclear p40-NRF2 expression (highest quartile in Histoscore) predicted poor outcome within the patients with M0 disease at the time of diagnosis.

A high nuclear NRF1 immunohistochemical expression in pigment cells correlated with a worse survival (*p* = 0.048) in patients without nodal metastases at the time of diagnosis (*n* = 45, Figure 6(a)). When N1-3 cases were considered, nuclear NRF1 had no prognostic significance (*p* = 0.72, Figure 6(b)). When analysing the NRF2 expression in patients with M0 disease at the time of diagnosis (*n* = 71, Figure 6(c)), we found that the highest quartile of the nuclear NRF2 expression in pigment cells correlated with a significantly worse survival rate (*p* = 0.033). mRNAs or miRNAs had no prognostic significance. In multivariate analysis, neither of these variables exceeded the prognostic power of Breslow thickness.

## 4. Discussion

In this work, we studied for the first time the protein level of the redox-sensitive transcription factor NRF1 together with NRF2. Very early on in melanoma carcinogenesis, both NRF1 and NRF2 were found to be downregulated at the protein level as well as at the mRNA level. The results of the mRNA expression from our own patient cohort are further supported by analyses from three independent melanoma patient sample sets from the GEO database [16–18]. We also studied the expression of some redoximiRNAs from the same sample set and described some new data on their expression level changes in melanoma carcinogenesis and correlation with immunohistochemical and mRNA expression levels of NRF1 and NRF2.

### 4.1. NRF1 and NRF2 in Melanoma

We carefully examined the expression of NRF1 with two different antibodies targeting the N-terminal and C-terminal sites of the protein. As NRF1 binds the ER membrane with its N-terminal domain and is cleaved upon activation [21], it is logical that the antibody targeting the N-terminus showed a perinuclear staining pattern under light microscopy and a strong expression in the membranous fraction in the immunoblot. By contrast, the antibody recognizing the C-terminus showed predominantly a nuclear staining pattern and a strong expression in the nuclear fraction in the immunoblot resembling active NRF1. In immunohistochemistry, the protein level of NRF1 had a decreasing trend during melanoma carcinogenesis. We also observed that the NRF1 mRNA level decreased from benign naevi to dysplastic naevi and to melanomas and that their levels associate with ulceration. There is human sample set data showing that NRF1 mRNA levels are also downregulated in prostate carcinoma [22] but they are upregulated in oesophageal squamous cell carcinoma [23]. Conditions such as oxidative stress, proteasomal inhibition, ER stress, and hypoxia activate NRF1 to function as a transcription factor [4]. An early increase of ER stress in melanomas and adaptation to it as a driver of malignancy was discussed a decade ago [24]. Therefore, the dysregulated NRF1 might be a mere surrogate marker for more robust biological processes behind the pigment cell malignancy, but it can also play a significant role in carcinogenesis, as its deficiency can lead to genomic instability [25]. Indeed, there is some experimental evidence from a study using keratinocyte cell culture, mouse model, and patient samples that NRF1 functions as a tumor suppressor in the skin by activating DNA damage repair after ultraviolet (UV) B irradiation and is downregulated in human squamous cell carcinoma compared to normal skin [26]. Nuclear NRF1 expression, which predicted exceptionally poor melanoma-specific outcome in those patients without nodal metastases at the time of diagnosis, might benefit the carcinogenetic process by alleviating oxidative and ER stress accumulated in the aggressive disease [24]. To the best of our knowledge, there is no further published data on the prognostic role of NRF1 in cancers yet, except for our recent report pointing out that low nuclear and high cytoplasmic NRF1 is associated with poor overall survival in diffuse large B-cell lymphoma [27].

An elevated protein expression level of NRF2 has been noted in solid cancers as a prognostic feature, as has been summarized in a previous meta-analysis [28]. Alterations of NRF2 mRNA in different cancers have been reported, for example, its decrease in breast and oesophageal squamous cell carcinoma compared to normal tissue [23, 29]. We have described the prognostic role of NRF2 in a melanoma sample set recently [12, 13] and reported that the NRF2 expression increased from benign to dysplastic naevi to primary and metastatic melanomas. While those studies were rather hampered by the unspecific antibody against NRF2 (clone C-20), as also discussed in detail in another study [30], here, we used an antibody against NRF2 that is phosphorylation-specific. Phosphorylation of the amino acid serine in position 40 by protein kinase C in response to oxidative stress dissociates NRF2 from its inhibitor Keap1, promoting its translocation into the nucleus [14]. Thus, the signal detected with this antibody represents an active transcription factor and is mainly seen in the nuclei with a decreasing trend of expression in melanoma compared to nonmalignant lesions. The differences in the expression trends seen between the previous and the current report could be explained by the specificity of these two antibodies. Despite the contradictory result in the expression trends, current p40-NRF2 results support our previous observation, namely, that the NRF2 expression favours worse disease-specific survival, and the role of NRF2 in melanoma carcinogenesis seems to be rather consistent with NRF1.

### 4.2. MicroRNAs in Melanoma

We described the expression of seven different miRNAs in our naevi and melanoma sample set which was selected based on their NFE2L family and redox association. Expression levels of miR-93 and miR-340 increased significantly from all naevi to primary melanomas and metastases. Apart from NRF2, miR-93 associates with lung cancer proliferation, migration, and invasion *in vitro* and is upregulated in multiple cancers [31, 32]; our data complements this background. The miR-340 is also described to regulate the master regulator of melanocyte development and melanoma progression, microphthalmia-associated transcription factor (MITF) [33], and MAPK-signalling by modulating the expression of multiple components of this pathway *in vitro*. Our data is in line with these findings, since miR-340 expression levels were significantly elevated in several tested melanoma cell lines compared to normal human epidermal melanocytes [34].

Based on the available literature, miR-510 may have either cancer promoting or suppressing properties, depending on the cancer type. Overexpression of miR-510 can increase cell growth and migration as well as invasion and colony formation of breast cancer *in vitro* [35], while the effect is just the opposite in renal cell carcinoma [36]. In ovarian cancer, high miR-510 expression associates with early stage and predicts prolonged survival [37, 38]. In our material, miR-510 expression strongly correlated with the presence of lymph node metastases at the time of diagnosis and, on the other hand, showed a decreasing expression from all naevi to primary melanomas and further to metastases. Similarly, primary gastric cancers were found to have higher miR-510 expression than lymph node metastases [39].

### 4.3. Correlation of miRNAs with NRF1 and NRF2

The posttranscriptional regulation of gene expression by imperfect matching of miRNA leads to the inhibition of mRNA translation and eventually to mRNA degradation [6], and therefore, the effect of miRNA would be generally negative when seen typically on a protein level. Thus, it is logical that miRNA levels correlate negatively with protein levels rather than mRNA levels. From the studied miRNAs, miR-23b-3p, miR-93-5p, and miR-144-3p are predicted inhibitors of NRF2 mRNA, miR-212-3p of both NRF1 and NRF2 mRNA, and miR-340 of NRF1 mRNA, based on the miRmap database [40]. The miR-383 and miR-510 were not predicted to bind NRF1 or NRF2 mRNA. In our material, only miR-23b-3p correlated positively with NRF2 mRNA in the whole material and significantly with NRF2 immunohistochemical expression in malignant cases. Interestingly, according to this database, miR23b-5p, the complementary sequence of the same miRNA hairpin structure, would be an inhibitor to NRF1. miR-340 negatively correlated with both nuclear NRF1 and p40-NRF2 protein expression. Additionally, miR-510 correlated negatively with the expression of nuclear NRF1 in malignant samples. Although miR-93-5p, miR-144, and miR-212 had a predicted relation with NRF1 and NRF2, apparently, this is not the case in melanoma and the lack of correlation may reflect the general discoordination within a cancer cell.

### 4.4. miRNAs in respect to Clinical Variates

miR-144 and miR-212 associated positively with melanoma ulceration, a highly important prognostic and predictive factor of melanoma. Previously, let-7b-5p, miR-16, miR-106b, and miR-137 were described to be associated with melanoma ulceration that can be linked to anchorage-independent growth, aggressive disease, and progression [41–44]. Also, miR-212 and miR-340 associated with pigmentation. The association of miR-340 to pigmentation could be explained by the relation to melanocyte differentiation regulator MITF [33]. Other pigmentation-related miRNAs reported are miR-16, miR-125b, miR-155, miR-203, miR-204, and miR-211 [45–49]. In univariate analysis, mRNAs or miRNAs had no prognostic significance, possibly due to the small amount of tested primary melanomas (*n* = 17‐20).

Although the current study addresses for the first time the association of NRF1 in melanoma, its retrospective nature causes also some weaknesses. In particular, despite the material was sufficient to produce the current results, the size of effect may have been different with the larger sample size. Moreover, we did not have data on ethnicity, UV exposure, skin type, or the number of blistering sunburns available, which is a confounding factor in the study.

## 5. Conclusions

This data suggests that there is a loss of NRF1 and NRF2 mRNA and protein levels during different stages of melanoma carcinogenesis. This early change can be seen between the groups of benign and dysplastic proliferative naevi that are known to harbour oncogenic mutations [50]. High nuclear NRF1 and NRF2 protein expression may also predict a dismal outcome in patients before nodal or distant metastases occur, respectively. Thus, it is plausible that even if these redox-regulating and stress-sensing transcription factors have a protective role against melanoma carcinogenesis, they can be exploited as tumor-progressing factors in the malignant phase, as suggested earlier in other tumor types [51]. Additionally, redoxmiRs miR-144, miR-212, and miR-510 appear to associate with aggressive melanoma features, and their possible prognostic value should be evaluated in larger cohorts.

## Figures and Tables

**Table 1:** Patient cohort.

			IHC analysis	RNA analysis
Total number of patients			172	54
Age median (years)			60	67
Samples per diagnosis	Compound naevus		25	5
	Intradermal naevus		28	4
	Dysplastic naevus		46	4
	Nodular melanoma		48	15
	Superficially spreading melanoma		32	5
	Acral melanoma		9	1
	Metastasis		67	20
Number of patients with malignant melanoma			68	21
	Median age (years)		70	71
	Males		53	14
	Females		15	7
	Ulceration		19	11
	No ulceration or not defined		49	10
	Breslow	≤1 mm	11	1
		1–1.9 mm	20	0
		2–3.9 mm	13	3
		>4 mm	24	17
	Breslow mean		3.6 mm	9.2 mm
	Breslow median		2.5 mm	6.0 mm

**Table 2:** Antibodies used in immunohistochemical staining and western blot.

Immunohistochemistry								
Antigen	1^st^ antibody	Dilution	Revealing of antigen	Incubation	Detection of 1^st^ AB	Colour development	Product	Clone
NRF1	Rabbit polyclonal anti-NRF1 (C-terminal)	1 : 500	Tris-EDTA pH 9.0	2 h RT	Dako EnVision	DAB	Sigma, HPA065424	Not reported
NRF1	Mouse monoclonal anti-NRF1 (N-terminal)	1 : 500	10 mM sodium citrate buffer pH 6.0	45 min RT	Dako EnVision	Fast Red	Santa Cruz Biotechnology, sc-365651	E-4
NRF2	Rabbit monoclonal anti-NRF2 phospho S40	1 : 600	10 mM sodium citrate buffer pH 6.0	30 min RT	Dako EnVision	DAB	Abcam, 76086	EP1809Y
Western blot								
Antigen	1^st^ antibody	Dilution					Product	Clone
NRF1	Rabbit polyclonal anti-NRF1 (C-terminal)	1 : 2500					Sigma, HPA065424	Not reported
NRF1	Mouse monoclonal anti-NRF1 (N-terminal)	1 : 1000					Santa Cruz Biotechnology, sc-365651	E-4
NRF2	Rabbit monoclonal anti-NRF2 phospho S40	1 : 10000					Abcam, 76086	EP1809Y
*β*-Actin	Mouse monoclonal anti-*β*-actin	1 : 5000					Novus Biologicals, NB600-501SS	AC-15
PCNA	Mouse monoclonal anti-PCNA	1 : 2000					Cell Signalling Technology, #2586	PC10
	HRP-conjugated goat-anti-mouse	1 : 5000					Santa Cruz Biotechnology, sc-2055	—
	HRP-conjugated goat-anti-rabbit	1 : 5000					Santa Cruz Biotechnology, sc-2054	—

RT = room temperature, DAB = diaminobenzidine.

**Table 3:** Primers used in qPCR.

RNA target	Product company	Primer sequences	Amplicon length (base pairs)	Function related to oxidative stress (references)
mRNA NRF2	Sigma	Forward: 5′-CAATGAGGTTTCTTCGGCTACG-3′ Reverse: 5′-AAGACTGGGCTCTCGATGTG-3′	72	Major redox response regulator [1]
mRNA NRF1	Sigma	Forward: 5′-ATGGAAATGCAGGCCATGGAAG-3′ Reverse: 5′-GAGGGGCACTGTACAGGATTT-3′	61	Redox response regulator [5]
GAPDH	Sigma	Forward: 5-TGGAAGGACTCATGACCACA-3′ Reverse: 5-CCATCACGCCACAGTTT-3′		—
miR-23B-3p	Qiagen	5′AUCACAUUGCCAGGGAUUACC		Predicted NRF2 inhibition [40]
miR-93-5p	Qiagen	5′CAAAGUGCUGUUCGUGCAGGUAG		Predicted NRF2 inhibition [40]
miR-144-3p	Qiagen	5′UACAGUAUAGAUGAUGUACU		Predicted NRF2 inhibition [8, 40, 52]
miR-212-3p	Qiagen	5′UAACAGUCUCCAGUCACGGCC		NRF1 and NRF2 inhibition, interaction with Mn-SOD
miR-340-3p	Qiagen	5′UCCGUCUCAGUUACUUUAUAGC		MAPK signalling, predicted NRF1 inhibition [40]
miR-383-5p	Qiagen	5′AGAUCAGAAGGUGAUUGUGGCU		No predicted inhibition of NRF1/NRF2
miR-510-5p	Qiagen	5′UACUCAGGAGAGUGGCAAUCAC		No predicted inhibition of NRF1/NRF2
RNU-6B	Qiagen	(Not reported, product no. 218300 cat. no. MS00014000)		—

## Data Availability

The data used to support the findings of this study are available from the corresponding author upon request.

## Supplementary Materials

Supplementary Materials Supplementary Table 1: association between variables and diagnoses.
