# Supplementary material for: NRF1 and NRF2 mRNA and Protein Expression Decrease Early during Melanoma Carcinogenesis: An Insight into Survival and MicroRNAs
Source: Oxid Med Cell Longev. 2019 Sep 4;2019:2647068. doi: 10.1155/2019/2647068 (PMC6794976; doi:10.1155/2019/2647068)
Supplement: Supplementary Materials — Supplementary Table 1: association between variables and diagnoses. [file 2647068.f1.docx]

|  | From benign to dysplastic naevi | From benign naevi to dysplastic naevi and malignant primary melanoma | From all naevi to malignant primary melanomas | From all naevi to malignant primary melanomas and metastases | From malignant primary melanomas to metastases |
| --- | --- | --- | --- | --- | --- |
| NRF1 n IHC | <0.001 | <0.001 | <0.001 | <0.001 | 0.012 |
| NRF1 sp IHC | NS | <0.001 | <0.001 | <0.001 | NS |
| NRF2 IHC | 0.005 | 0.018 | 0.050 NS | 0.002 | NS |
| NRF1 mRNA | 0.034 | 0.066 NS | NS | NS | NS |
| NRF2 mRNA | 0.050 NS | 0.012 | 0.049 | NS | NS |
| miR-23B | NS | NS | NS | NS | NS |
| miR-93 | NS | 0.013 | 0.009 | 0.023 | NS |
| miR-144 | NS | NS | NS | NS | NS |
| miR-212 | NS | NS | NS | NS | NS |
| miR-340 | NS | 0.026 | 0.024 | 0.045 | NS |
| miR-383 | NS | NS | NS | 0.024 | NS |
| miR-510 | NS | 0.052 NS | 0.045 | 0.002 | NS |

Supplementary table 1. Association between variables and diagnoses
